# Surgery

**Published:** 1878-07

**Authors:** 


### SURGERY.

ON A CASE OF UN-UNITED FRACTURE.—Dr. Max Schüller, of Griefswald. (*Deutsche Medicinishe Wochenschrift*, March 2, 1878.)

Dr. Schüller observes that the employment of antiseptic precautions must materially change the opinions formerly entertained as to the danger of resecting the ends of the fragments in un-united fracture. Hence, of late years, we find this mode of treatment very frequently resorted to, amongst others by Volkmann, Bardeleben, and Langenbeck. The value of this method of treating false joint, as compared with the many others from time to time practiced, is, the author considers, yet to be determined. As a partial solution of the question, Dr. Schüller has published this case.

Frau H., 40 years of age, a healthy vigorous woman, sustained a fracture of the leg from the wheel of a heavily laden wagon passing over it. Gypsum bandages were immediately applied, but, after eight weeks' treatment, no consolidation had taken place, and no formation of callus could be felt. A fresh trial of plaster of Paris bandage was now made, and a three per cent. solution of carbolic acid was injected from a hypodermic syringe near the seat of fracture; a plan recommended of late years by Professor Hueter for the treatment of cases of delayed or nonunion. Six months were consumed in various trials, and, at the end, there was not the slightest degree of union. An operation was then decided upon. A careful examination showed a marked interval between the fragments, so that pegging was not thought suitable. An incision, two and a half inches long, was therefore made, the seat of fracture exposed, and the periosteum raised from the ends of the bone, which were rounded, while between them passed the tendinous termination of the tibialis anticus muscle. There was not a trace of bone-proliferation. The muscle was now restored to its proper position, and the ends of the bones cut off, partly with the saw, and partly with the chisel, to the extent of a centimetre from each. After forcible traction, the fresh surfaces of bone could be accurately adjusted together, the fibrous connection of the broken ends of the fibula being divided simply with the knife, but the bone not otherwise interfered with. As the fragments of the tibia showed no tendency to separate, ivory pegging, or silver-wire suture, was not employed, but the carefully preserved periosteum was accurately united by fine cat-gut sutures, a small opening only being left to allow a fine drainage-tube to pass between the bones. Another and larger drain was passed outside the periosteum, and through a counter-opening made in the calf. The skin-wound was now sutured with cat-gut, the limb enveloped with salicylized jute, and a gypsum bandage, reaching over the knee, was applied. The whole operation was performed with strict antiseptic precautions. Except the day afterwards, when the temperature rose to 38.5° C. (101.3° F.), there was never any fever. The whole dressing, plaster of Paris bandage included, was changed every few days at first; then a window was made in the plaster, and the dressings of the wound alone renewed. The external wound healed by first intention, but a slight discharge continued for a long time from the interior. A small fragment of bone subsequently exfoliated, and then immediate and thorough healing followed; and, at the end of four months, complete consolidation had taken place, in good position, and with a shortening of only half a centimetre at the outside. The author preserved the periosteum in this case with the greatest care, believing it has the chief part in the act of union, the endosteum somewhat less, and the bonesubstance proper very little. The plan of pegging, he considers, has its principal result in fixing the fragments. He believes little in its power of exciting proliferation of bone when employed under antiseptic precautions, which accounts, in his opinion, for several recent failures of this method. In cases, therefore, where resection of the ends of the bone is indicated, he would perform the operation subperiosteally, accurately unite the margins of the divided periosteum, and only use pegs where necessary to maintain the fragments in a position, and at rest.

Transplantation of the Urethra to the Perineum.—In the *Archiv. für Heilkunde*, Heft vi., 1877, a case of cancer of the penis is reported, in which Professor Theirsch amputated that organ. After amputating at about the level of the pubic bone, and checking the hæmorrhage, a catheter was introduced into the urethra; an incision was then made along the raphé of the scrotum to the perineum, thus dividing the scrotum into two equal parts and exposing the urethra, the anterior termination of which was loosened from its attachment in the pubic arch for a distance of about four-fifths of an inch. A small incision was now made in the perineum between the posterior extremity of the scrotal wound and the anus, an inch and a half in front of the latter; the urethra was drawn through this opening, and its walls fastened in the borders of the wound. The operation was made under Lister's spray. Fourteen months after the operation the patient reported himself as perfectly free from all inconveniences. He could project his urine forward at angle of 45 deg. from the vertical line.

Exophthalmic Goitre cured by Galvanization of the Sympathetic.—Dr. Ancona (*Giornale Veneto delle Scienze Mediche*) relates the case of a young girl aged 19, of habitually bad health, who suffered from exophthalmos and goitre. She was emaciated, weak, suffered from diarrhœa and frequent flushings of the face; was irritable and capricious, and unceasingly dyspeptic. Dr. Ancona proposed galvanization of the first cervical ganglia of the sympathetic. The poles of a Stöhrer's battery were applied on each side of the neck, behind the angle of the jaw, pressing backwards the sterno-mastoid muscles. A current of ten elements was passed for a time varying from three to five minutes. After a few days, the circuit was frequently interrupted. The physiological effects observed were the following: dilatation of the pupil each time the circuit was closed, more marked on the side of the negative pole; slight contractions of the sterno-mastoid; scalorrhœa, with a taste of copper in the mouth; sometimes giddiness. At the end of five months, a hundred electrizations had been applied and very well borne. Arsenical treatment was added. From the beginning of the application of electricity, there was notable amelioration, and at the end of five months the state of the patient was very satisfactory. Her weight had increased by 30 lbs. Her face and mucous membranes resumed their normal color; her eyelids regained their mobility; the thyroid gland diminished in volume; the arterial pulsation ceased to be visible; the impulse of the heart became regular; the pulse fell; menstruation became regular; digestion was restored; and strength returned.

Treatment of Vaginismus.—Dr. F. Weber, of St. Petersburg (*Allg. Med. Central Zeitung*, Nos. 1 and 2, 1878, and *Allg. Wien. Med. Zeitung*, January 22, 1878,) thinks that the treatment of vaginismus should depend upon its cause, and should not be the same in all cases. Some local cause should always be sought for, and both local and general treatment are advisable.

The most common causes of the affection, he thinks, are a rigid condition of the hymen, gonorrhœal or catarrhal inflammation of the vagina, and also cicatrices, ulceration, or excoriation of the vulva and outer parts of the vagina.

Organic contraction should be treated by methodical dilatation, at first with compressed sponge, and subsequently with Fergusson's specula, the size of which should be gradually increased. An ointment of belladonna is of great service at the same time. Inflammation of the vagina should be treated with cloths, wet with a solution of sugar of lead, injections with or without opium, and belladonna suppositories. In the later stages, cauterization, with a solution of nitrate of silver, gives excellent results. This is especially serviceable when there are excoriations. Warm hip baths lessen the irritability of the nerves, and are of service. In addition to the local treatment, tonics and nervines should be used—especially bromide of potassium, iron and valerian. When no local trouble is to be found, and the sufferings of the patient are very severe, division of the nervus pudendus, as recommended by Simpson and Sims, should be practiced. The removal of the hymen itself or the myrtiform caruncle, Weber has never found necessary (though it has been repeatedly done by Sims and others).

Orthopædic Treatment of Paralysis of Ocular Muscles. (*Wien. Med. Wochenschrift*, No. 13, 1878.)

The following method based on the principle of passive move ments, is recommended by Michel. (klin. Monatsbl, f. Augenheilk, Nov., 1877):

The conjunctiva is seized over the insertion of the paralyzed muscle with an ordinary forceps, and the eye is pulled in the direction of that muscle, so as to imitate and in fact exceed the contraction of the paralyzed muscle. This is repeated two to three times. There is an immediate gain in strength of the muscle, which however, is not permanent. Nevertheless the duration of treatment is shortened and the contracture of the antagonistic muscle is avoided.

Treatment of Varicose Veins.—(*Mittheil. des Wiener Med. Doctoren-Collegiums*, No. 8, 1878.) In one of the numbers of the *Transactions* of the Wiener Medicinshe Doctoren-Collegiums for the latter part of 1877, Dr. Englisch published a paper on the treatment of hernia by injections of alcohol. The favorable results which he obtained from this mode of treatment in hernia induced him, he states, to test its value as a method of radically curing varicose veins. He states that this method for the radical cure of varices has the advantage over all others that it is perfectly harmless.

The method which Dr. Englisch pursues is very simple. The vein and a fold of the skin are caught up between the thumb and finger, and a needle of a Pravaz syringe is inserted in such a way that its point shall be immediately behind the vein. The contents of the syringe, from one to one and a half cubic centimetres of a fifty per cent. sample of alcohol, are then discharged in the immediate neighborhood of the vein. A small knot forms at the point of injection, and very often there is a momentary appearance of contraction in the veins. On the third day, there will be a considerable infiltration at the point of injection, which differs according to the irritability of different persons. In individuals who were very irritable, there was considerable redness produced, and in four or five cases suppuration ensued. The suppuration was only in the *neighborhood* of the vein, however; the vessel itself remained sound and healthy. The abscesses were as large as a bean, but gave rise to no trouble whatever. In none of Dr. E's cases was there any rise of temperature, though he examined carefully with reference to this point. When the infiltration softened and the swelling subsided, a change in the veins themselves became apparent. They were much smaller and harder at the point of injection and its vicinity, and felt like hard cords; and at the sides of these cords very well-marked grooves could be felt.

In a few of these cases, a single injection sufficed to cause a complete cure; but in the majority of cases, Dr. E. found it necessary to make three or four, and in one person he made as many as ten injections in both limbs.

The results are most favorable when the affected veins are spread out in the form of a plexus, and the cases are most difficult to treat when the varicose vessels give off a number of branches. The subjective troubles in consequence of the operation are very slight, and only require rest of the limbs.

The duration of treatment varies. Even in those cases where a complete cure cannot be obtained, the efficiency of the palliative means of treatment will be rendered much greater.

The mode of treatment is perfectly harmless, and if it does not succeed, the other procedures may then be resorted to.

ANTISEPTIC TREATMENT OF BURNS.—Bush. (*Centralbl. f. Chir.*, No. 14, 1878.)

At the last congress of German surgeons, W. Bush detailed his treatment of burns with Lister's antiseptic method. The burn is cleansed with a solution of carbolic acid; during the time of bandaging carbolic acid spray is constantly kept up. The wounds are thereupon covered with an ointment of boric acid spread on linen. Upon this an ordinary Lister's dressing is placed. If the burns are not very extensive, the bandage can remain in place for a considerable time. The growth of granulations is thus checked; and but little pus is produced while the cicatrization proceeds rapidly. The subsequent cicatrix is smooth and elastic. (Lister himself uses a similar method.)

